# Characterisation of infection-induced SARS-CoV-2 seroprevalence amongst children and adolescents in North Carolina

**DOI:** 10.1017/S0950268823000481

**Published:** 2023-04-03

**Authors:** Amina Ahmed, Michael E. DeWitt, Keerti L. Dantuluri, Paola Castri, Asare Buahin, William H. LaGarde, William S. Weintraub, Whitney Rossman, Roberto P. Santos, Michael Gibbs, Diane Uschner, for the COVID-19 Community Research Partnership

**Affiliations:** 1 Levine Children’s Hospital, Atrium Health, Charlotte, NC, USA; 2 Department of Internal Medicine, Section on Infectious Diseases, Wake Forest University School of Medicine, Winston-Salem, NC, USA; 3 Milken School of Public Health, George Washington University, Washington, DC, USA; 4 Department of Pediatrics, WakeMed Health and Hospitals, Raleigh, NC, USA; 5 MedStar Healthcare Delivery Research Network, MedStar Health Research Institute, Washington, DC, USA; 6 MedStar Healthcare Delivery Research Network, Georgetown University, Washington, DC, USA; 7a Center for Outcomes Research and Evaluation, Atrium Health, Charlotte, NC, USA; 7b Department of Emergency Medicine, Atrium Health, Charlotte, NC, USA; 8 University of Mississippi Medical Center, Jackson, MS, USA

**Keywords:** Adolescent, child, COVID-19, paediatrics, SARS-CoV-2, seroepidemiologic studies, serology

## Abstract

Few prospective studies have documented the seropositivity among those children infected with severe acute respiratory syndrome coronavirus 2. From 2 April 2021 to 24 June 2021, we prospectively enrolled children between the ages of 2 and 17 years at three North Carolina healthcare systems. Participants received at least four at-home serological tests detecting the presence of antibodies against, but not differentiating between, the nucleocapsid or spike antigen. A total of 1,058 participants were enrolled in the study, completing 2,709 tests between 1 May 2021 and 31 October 2021. Using multilevel regression with poststratification techniques and considering our assay sensitivity and sensitivity, we estimated that the seroprevalence of infection-induced antibodies among unvaccinated children and adolescents aged 2–17 years in North Carolina increased from 15.2% (95% credible interval, CrI 9.0–22.0) in May 2021 to 54.1% (95% CrI 46.7–61.1) by October 2021, indicating an average infection-to-reported-case ratio of 5. A rapid rise in seropositivity was most pronounced in those unvaccinated children aged 12–17 years, based on our estimates. This study underlines the utility of serial, serological testing to inform a broader understanding of the regional immune landscape and spread of infection.

## Introduction

The mortality rate of acute coronavirus disease 2019 (COVID-19) has typically followed a strong age gradient with children being at lower risk of severe clinical outcomes [1–5]. However, a growing body of evidence supports the finding that the seroprevalence of severe acute respiratory syndrome coronavirus 2 (SARS-CoV-2) among children and adolescents is much higher than previously thought [6, 7]. While infections have been shown to be milder in children, children infected with SARS-CoV-2 are still susceptible to severe outcomes such as those associated with multisystem inflammatory syndrome (MIS-C) [8]; some portion of those infected go on to experience post-acute COVID-19 sequala with studies indicating 4% of seropositive children reporting at least one symptom for longer than 12 weeks [9]. Children also play a major role in the transmission of the virus [10, 11], and thus understanding the true burden of disease in this section of the population is crucial.

The United States National Commercial Laboratory Seroprevalence Survey has monitored infection-induced seroprevalence in all age groups since August 2020, with a recent focus on children aged ≤17 years [7, 12, 13]. The median estimated seroprevalence of infection among children from eight states increased from 8% in August 2020 to 37% in May 2021 [6]. Based on these data, Couture and colleagues, in a cross-sectional analysis of residual serum samples, estimated 5–9 times more SARS-CoV-2 infections in children than reported by case-based surveillance [6]. Although these surveys highlight the prevalence of SARS-CoV-2 infections among children, they are limited by their reliance on residual clinical specimens that may not be representative of the general population [6, 7, 13].

We describe trends in prevalence of infection-induced antibodies using serial at-home serological testing in a cohort of 2- to 17-year-old children enrolled in a prospective, syndromic surveillance study in North Carolina. We used seroprevalence estimates to calculate infection-to-reported case ratios for a better understanding of the local burden of SARS-CoV-2 infections.

## Materials and methods

### Study design and participants

The COVID-19 Community Research Partnership (CCRP) is a multi-site, prospective cohort study combining electronic symptom surveillance with serological surveillance [14, 15]. From 2 April 2021 to 24 June 2021, we enrolled children between the ages of 2 and 17 years at three North Carolina healthcare systems based in the cities of Charlotte, Winston-Salem, and Raleigh. Caregivers consented and participants aged ≥13 years assented electronically. Caregivers provided demographic information at enrolment. From enrolment through 31 December 2021, participants received daily electronic surveys soliciting symptoms of COVID-19-like illness and receipt of COVID-19 vaccines. Additionally, participants were mailed four or more serology tests to be completed once monthly using fingerprick blood. A smartphone application was used to upload results. Testing was completed by 31 October 2021.

### Ethics approval

This study was approved by the institutional revenue board for Wake Forest University School of Medicine (IRB00064912).

### Antibody detection

An Innovita Biological Technology SARS-CoV-2 lateral flow assay (Beijing, China) was used to detect SARS-CoV-2 immunoglobulin G (IgG) with a sensitivity of 84.5% and a specificity of 99%; the assay detected but did not differentiate between anti-spike and anti-nucleocapsid IgG. Infection-induced antibodies were defined as the presence of a positive IgG prior to any reported vaccine dose.

### Statistical analysis

To estimate seroprevalence from infection over time, a Bayesian framework was employed accounting for assay sensitivity and specificity. Participants from North Carolina and South Carolina were pooled for the analysis. The proportion of respondents with infection-induced antibodies was estimated for each study month. To estimate the North Carolina seroprevalence from infection using the cohort data, multilevel regression with poststratification (MRP) was performed. A multilevel logistic regression was fitted with an intercept and random effects for age group in years [2–4, 5–11, 12–17], sex (male and female), and race (Black, White, and other), with adjustments for sensitivity and specificity of the assay [16]. Prevalence estimates were obtained by weighting the corresponding model estimates by the subgroup total using the National Center for Health Statistics Vintage 2020 Bridged-Race Postcensal Population Estimates [17]. Reported cases and vaccinations were retrieved from the North Carolina Health and Human Services dashboard [18]. Variant periods were defined as pre-Delta, Delta, and Omicron (pre-BA.4/BA.5), based on variant predominance in North Carolina following DeWitt et al. [19]. Infection-to-reported case ratios were calculated by dividing the estimated population seroprevalence for a 1-month timeframe by the cumulative reported cases for the prior month, in order to account for the delayed antibody response to a SARS-CoV-2 infection [20].

All analyses were conducted with R version 4.1.3 (2022-03-10) and Stan version 2.28.1 [21].

## Results

**Figure 1.**
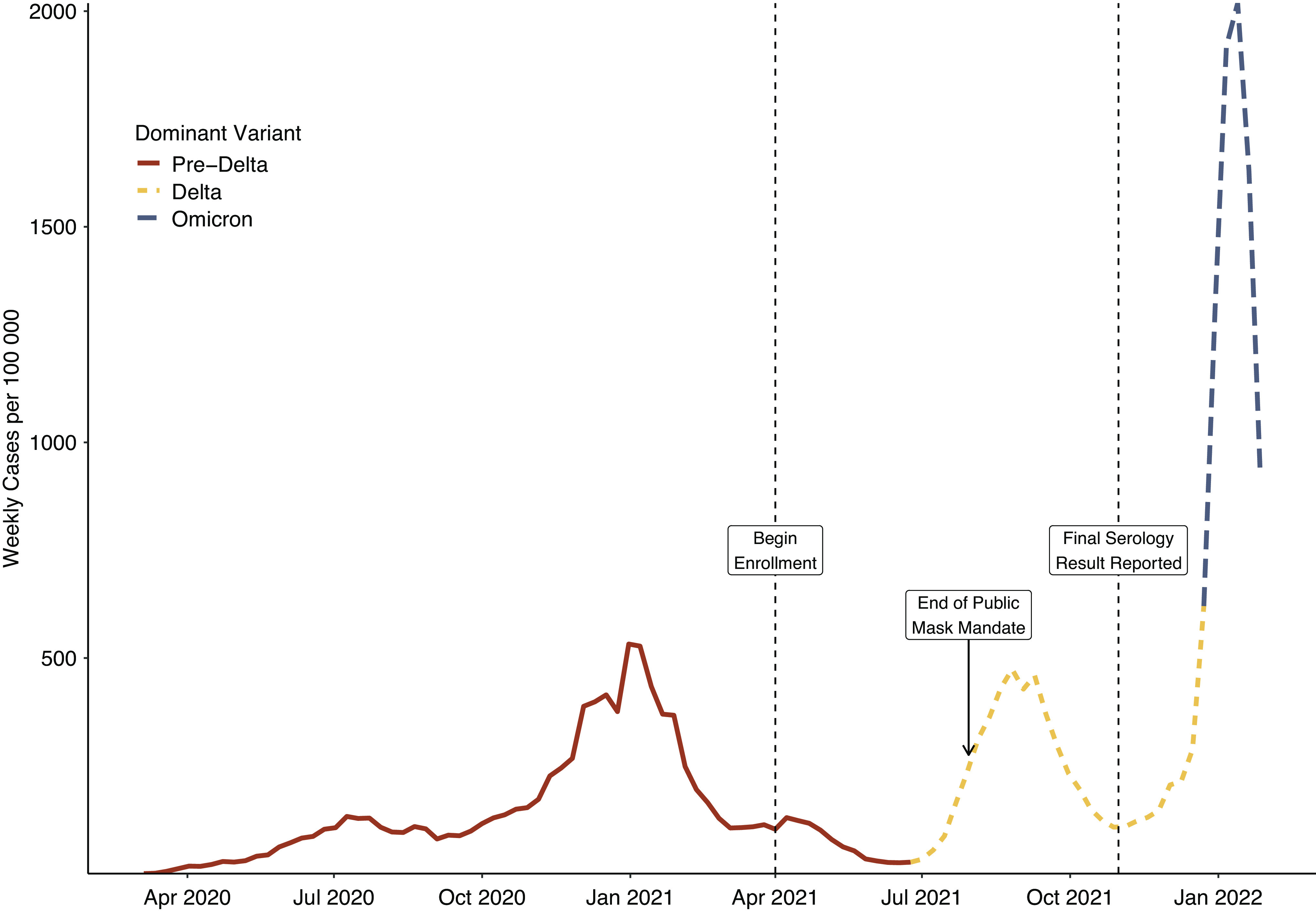
Reported COVID-19 weekly cases per 100,000 residents in the state of North Carolina from 1 March 2020 to 1 February 2022, with the dominant variant represented.

**Figure 2.**
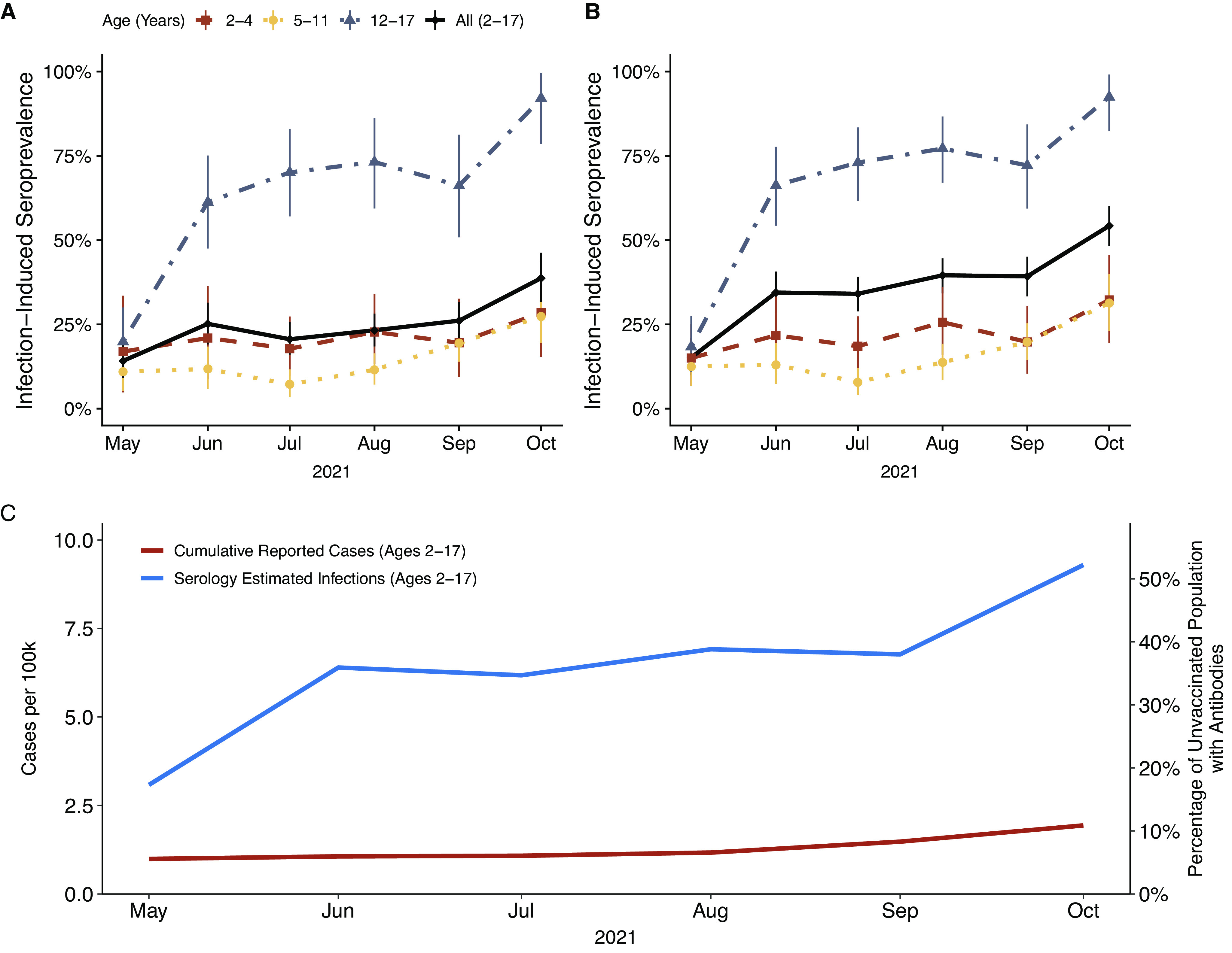
Estimated prevalence of infection-induced SARS-CoV-2 antibodies in study cohort overall and by age group (a). Estimated prevalence of infection-induced SARS-CoV-2 antibodies in North Carolina by age group estimated by multilevel regression with poststratification (b). All estimates are corrected for assay sensitivity and specificity and reflect 95% credible intervals. Comparison of reported SARS-CoV-2 cases with serology-estimated number of infections among North Carolina children aged 2–17 years who did not have any doses of vaccine (c).

**Figure 3.**
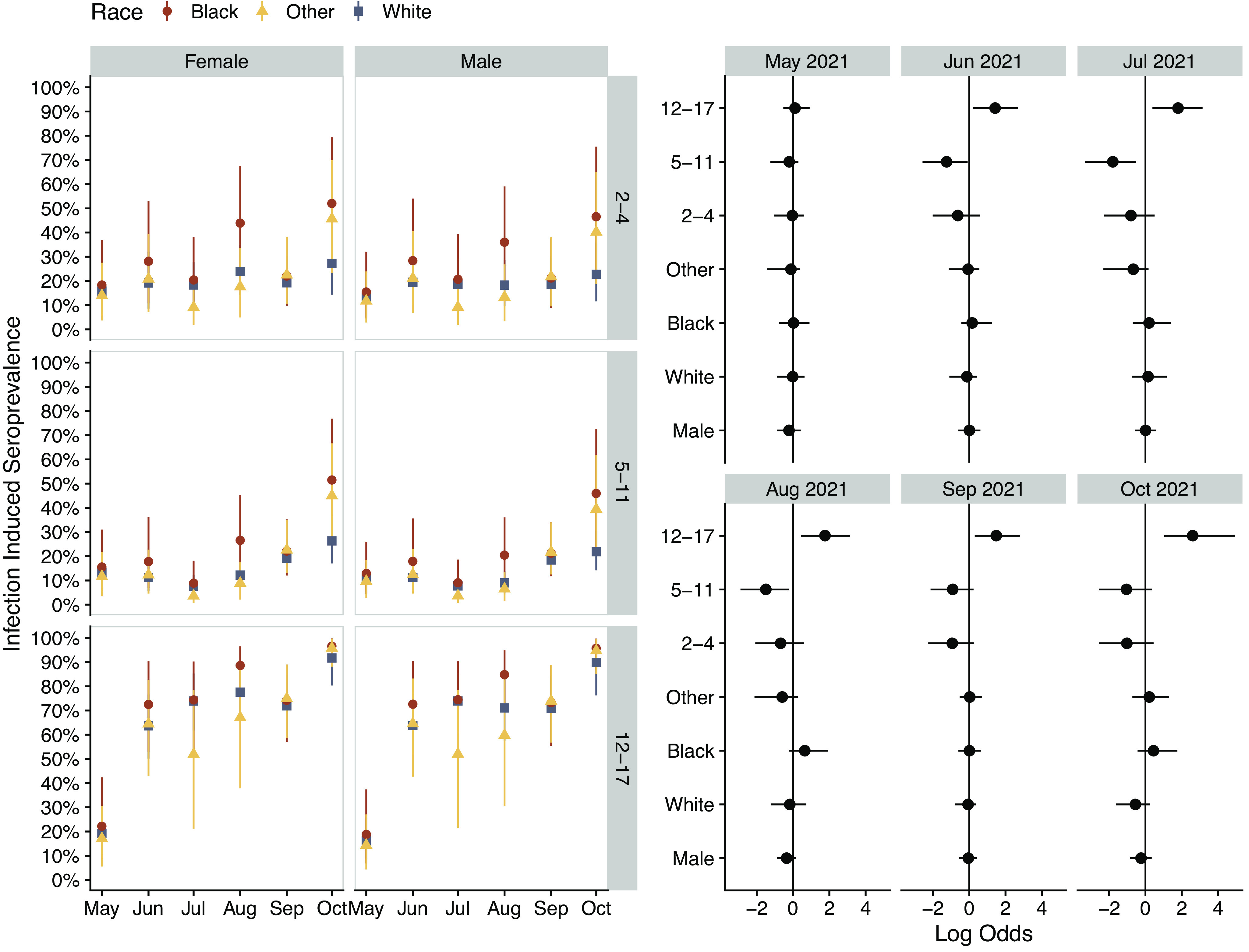
Modelled prevalence of infection-induced SARS-CoV-2 antibodies by age for North Carolina by demographic in 2021 (a). Simultaneous estimated model parameters (posterior log odds) for likelihood of seropositivity by month of data collection (b). Group level (random) effects are shown for race and age while the population (fixed) effect is shown for sex. All model estimates consider assay sensitivity and specificity and reflect 95% credible intervals.

A total of 1,058 participants from 35 and 6 counties in North Carolina and South Carolina, respectively, consented to both syndromic and serological surveillance. Participants were predominately White and non-Hispanic and returned a median of serology two tests (IQR 1–4) within a median of 32 days (IQR 27–38) (Table 1). During the study period, there was a substantial rise in community cases commensurate with the emergence of the Delta variant, with reported cases rising beginning in July 2021 and peaking in early September 2021 (Fig. 1). Seroconversion before vaccination occurred an average of 59.2 days after enrolment. Adjusting for sensitivity and specificity, the cohort infection-induced seroprevalence rose from 14.2% (95% credible interval, 9.2–19.8) in May 2021 to 38.7% (31.7–46.2) in October 2021 (Fig. 2a). MRP estimated infection-induced seroprevalence increased from 15.2% (9.0–22.0) in May to 54.1% (46.7–61.1) in October 2021 (Table 2), with the largest absolute increase occurring among the 12- to 17-year-olds. By October 2021, MRP estimated infection-induced antibody prevalence was 28.7% (15.8–43.7), 27.2% (19.5–36.1), and 92% (77.6–99.6) among unvaccinated children aged 2–4, 5–11, 12–17 years old, respectively (Fig. 2b). During this period, the estimated infection-to-case ratio increased from 3.1 (1.9–4.5) in May 2021 to a peak of 6.1 (5.1–6.9) in August 2021 before declining to 4.8 (4.1–5.4) in October 2021 (Fig. 2c and Table 2). In posterior estimates by unique cell, Black children less than 12 years of age tended to have point estimates of seroprevalence higher than any demographic in these age groups (Fig. 3a). Analysis of the regression coefficients suggests that those aged 12–17 years old were associated with a higher probability of infection-induced antibodies after June 2021 (Fig. 3b).

**Table 1.** Overall characteristics and key demographics

Characteristic	*N* = 1,058 (%)
Age (years), no. (%)	
2–4	146 (13.8)
5–11	495 (46.8)
12–17	417 (39.4)
Sex, no. (%)	
Female	537 (50.7)
Male	516 (48.8)
Unknown	5 (0.5)
Race, no. (%)	
Other	114 (10.8)
Black or African American	71 (6.7)
White	873 (82.5)
Ethnicity, no. (%)	
Hispanic or Latino	62 (5.9)
Not Hispanic/Latino	956 (90.3)
Not specified or unknown	40 (3.8)
Prior COVID-19 diagnosis (Y), no. (%)	93 (8.9)
Seroconverted prior to vaccination (Y), no. (%)	195 (18.4)

**Table 2.** Comparison of reported severe cute respiratory syndrome coronavirus 2 cases versus serology-based estimated infections and infection-to-case ratios among children in North Carolina

Month (2021)	Estimated seroprevalence (95% CrI)	Cumulative reported cases prior month^a^	Average estimated infections	Infection/reported case ratio (95% CrI)
May	15.2 (9.0–22.0)	99,046	308,500 (183,400–446,600)	3.1 (1.9–4.5)
June	34.4 (27.2–41.9)	106,251	639,700 (505,500–777,500)	6.0 (4.8–7.3)
July	34.2 (28.1–40.1)	108,016	620,100 (509,100–727,600)	5.7 (4.7–6.7)
August	40.5 (34.5–46.4)	116,979	708,300 (602,100–810,000)	6.1 (5.1–6.9)
September	39.2 (32.3–45.9)	147,831	676,200 (556,400–791,800)	4.6 (3.8–5.4)
October	54.1 (46.7–61.1)	193,676	926,600 (800,800–1,046,700)	4.8 (4.1–5.4)

Abbreviation: CrI, credible interval.

a Cumulative coronavirus disease 2019 cases reported by the North Carolina Department of Health and Human Services based on the prior month (e.g., seroprevalence estimate for May are compared to cases in April to allow time for development of IgG antibodies).

## Discussion

Our findings support the mounting evidence of a high seroprevalence of SARS-CoV-2 infection among children and adolescents [2, 3]. By October 2021, we estimate that more than 50% of children aged 2–17 years old in North Carolina had infection-induced antibodies. The greatest increase in seroprevalence occurred in the fall, coincident with the return to school and end of public masking mandates in North Carolina and South Carolina [22, 23]. Among adolescents aged 12–17 years who remained unvaccinated, the majority had infection-induced antibodies by June 2021, with an estimated 90% seropositive by October 2021. Despite increases in the availability of testing during 2021, the estimated infection-to-case ratios of 3.1–6.1 demonstrate that reported COVID-19 cases likely grossly underestimate the actual spread of infection.

The seroprevalence of infection-induced antibodies among children has exceeded that of adult age groups in the United States since November 2020 [1]. As of September 2021, the estimated seroprevalence was 30% (95% CI: 26–33%) among U.S. children aged 1–4 years, 38% (95% CI: 36–40%) among those aged 5–11 years, and 40% (95% CI: 38–41%) among adolescents aged 12–17 years [13]. In North Carolina, Couture and colleagues previously estimated ratios of SARS-CoV-2 infections to reported COVID-19 cases of 7.7 in August 2020, declining to 4.7 in May 2021 [6]. We report similar seroprevalence estimates for younger children as well as the continued underestimation of cases among children late in the second year of the pandemic. However, we estimated a much higher seroprevalence among adolescents by fall 2021. While national studies tend to report lower seroprevalence estimates than local studies [24], this variation may be also explained by important differences in study design. Like prior seroprevalence studies in children [25, 26], national surveys repeatedly assayed convenience samples of residual sera from vaccinated and unvaccinated children, with likely overrepresentation of children with increased access to or need for healthcare. In contrast, we conducted longitudinal surveillance in a more generalisable, community-based cohort, censoring for vaccination. Thus, our study more likely captured those who were asymptomatic or had mild infections and did not present for testing and thus would likely not have residual sera available for passive public health surveillance. Furthermore, many national cross-sectional surveys of residual sera employed serologic assays that detected only anti-nucleocapsid antibodies [7, 13]. It has been noted that not all children with SARS-CoV-2 infection develop these antibodies to nucleocapsid, and among those that do, the response may be short-lived compared with anti-spike antibodies [27, 28]. As such, our study likely captured more infections through the use of an assay detecting both anti-spike and anti-nucleocapsid antibodies. Lastly, the national surveys could not capture and thus could not stratify for important demographic variables known to impact seroprevalence estimates and infection-to-case ratios [3, 4].

We found that those unvaccinated adolescents aged 12–17 had the highest prevalence of infection-induced antibodies and further had several periods of large increases in seroprevalence. The estimated higher prevalence may be a product of differing contact patterns amongst this age group when compared with the younger age groups. Prior studies of contact patterns conducted in European countries have indicated that those aged 10–19 years have the highest number of reported contacts when compared to other age groups [29] – a pattern that was also seen in other high-income countries in a meta-analysis of contact pattern studies [30]. Understanding the role of contact patterns through the use of seroprevalence studies may help to shed light on the effectiveness of various non-pharmaceutical interventions or public health measures, especially when there is a high degree of asymptotic or pauci-symptomatic disease [31–33].

Likely the most profound change in contact patterns in children and adolescents during the study period was the widespread return to in-person education [34]. Due to varying masking requirements and mitigation measures employed by individual school districts, our study is unable to determine what proportion of the observed increase in seroprevalence was due to in-school transmission versus community transmission. Partnering with district and state educational leadership and with public health officials in North Carolina, the ABC Science Collaborative examined the impact of re-opening schools on the transmission of SARS-CoV-2 amongst children and adolescents [35]. In a study of in-school contact tracing between August 2020 and July 2021 in a private pre-kindergarten through 12th grade school, Thakkar and colleagues found that despite high rates of community incidence of SARS-CoV-2 infections, in-school transmission was limited when multilayered mitigation measures were in place, including masking, health screenings, and contact tracing [36]. Similarly, an analysis of United States–based, cross-sectional internet survey data from the COVID-19 Symptom Survey paired with reported case rates found that while in-person schooling increases the relative risk of COVID-19, these risks are attenuated or disappear depending on the number of school-based mitigation measures reported [37].

Our study highlights the opportunity to use Bayesian techniques with a relatively small number of prospectively enrolled participants conducting at-home testing, even among children and adolescents, to make small area inferences and better capture local disease dynamics for infections other than SARS-CoV-2. While traditional national sentinel-based frameworks leverage existing networks of healthcare providers, studies have shown that these frameworks may under-ascertain the disease dynamics in lower socio-economic populations who do not have access to these resources [38]. Minority recruitment and adjustment for underrepresentation are important for a more complete understanding of the impact a pathogen may have in a community and the associated interventions to improve health equity. For example, our study suggests higher rates of infection-induced antibodies amongst Black children younger than 12 years old, complementing similar studies with findings of higher prevalence of infection [26, 39] and MIS-C among children from minority racial groups [40]. Si and colleagues have shown how routine sampling of the IgG of hospital patients undergoing elective procedures can be used to complement existing state-based data in order to estimate levels of infection and vaccination derived immunity using similar Bayesian MRP frameworks [41]; however, a sampling of elective procedures likely fails to capture the immunology landscape amongst children and those from lower socio-economic backgrounds. Further still, without broader surveys, there is a risk that these blind spots to lower socio-economic population and children could be propagated forward in other modelling efforts [42], underlining the utility of studies such as ours.

Our study has a few limitations. The surveillance was limited to North Carolina and South Carolina, limiting generalisability of results. However, this geographic focus is important because public health measures may need to be deployed given local transmission dynamics, underlying comorbidities, and probability of adverse outcomes. For example, individuals in the Southeast United States are often disproportionately affected by many chronic diseases and infection [43, 44]. Our study recruitment strategy included email, advertisements, and social media posts, both in English and Spanish. Additionally, we established community-based partnerships with faith-based organisations to recruit minorities at community events. Despite these recruitment efforts, minorities were disproportionately underrepresented in our study, underscoring the importance of recruitment and retention strategies to improve representation. Prior studies have shown that minority groups, once enrolled, participate at rates similar to other racial and ethnic groups [14, 43]. A review of recruitment and retention strategies for low-income and minority populations indicates that language barriers, participant perceptions, and trust of the research were important considerations in improving representation in recruitment [45]. Different racial and ethnic groups appear to respond to different recruitment strategies, highlighting the likely need for a diversified approach to participant recruitment [46]. For future paediatric studies, recruitment strategies should utilise mixed approaches including the use of well-child visits or health fairs as an opportunity for study enrolment, especially in underrepresented communities [46].

Unlike prior national surveys [6, 7, 13], we were able to adjust for important variables such as race. Post-survey correction techniques and any small area estimates are ultimately limited by the representativeness of the survey respondents. Low response rates from a given demographic will result in higher levels of uncertainty in the inferences for those cells and will propagate to higher uncertainty in the overall estimates. Handling small cell sizes in a Bayesian framework will typically result in shrinkage towards the group mean rather than poststratification which can suffer from much higher variances [47], further underscoring the need for improved representation in studies. As this study ended prior to the emergence of the Omicron and subsequent variants, we are unable to make inferences on the role of specific variants on seropositivity. Finally, when estimating the infection-to-reported case ratios, we assumed that reported cases were amongst only those who were unvaccinated. This assumption could have the effect of reducing our effective susceptible population, biasing our infection-to-reported cases down.

## Conclusion

Although children have milder illness compared to adults with COVID-19, they play a major role in transmission, even if asymptomatic [11, 15]. Accurate estimates of the cumulative incidence of infection among children are therefore vital to understanding the impact on the larger community. By prospectively following a relatively small but regionally generalisable cohort for infection-induced antibodies, we were able to estimate the local burden of SARS-CoV-2 infections among children. As the pandemic evolves, similar unbiased regional serological surveillances can complement national surveys to elucidate the dynamics of SARS-CoV-2 infections in children and inform public health measures for individual communities and their associated immune landscapes.

## COVID-19 Community Research Partnership

Wake Forest School of Medicine: Thomas F. Wierzba, PhD, MPH, MS, John Walton Sanders, MD, MPH, David Herrington, MD, MHS, Mark A. Espeland, PhD, MA, John Williamson, PharmD, Morgana Mongraw-Chaffin, PhD, MPH, Alain Bertoni, MD, MPH, Martha A. Alexander-Miller, PhD, Paola Castri, MD, PhD, Allison Mathews, PhD, MA, Iqra Munawar, MS, Austin Lyles Seals, MS, Brian Ostasiewski, Christine Ann Pittman Ballard, MPH, Metin Gurcan, PhD, MS, Alexander Ivanov, MD, Giselle Melendez Zapata, MD, Marlena Westcott, PhD, Karen Blinson, Laura Blinson, Mark Mistysyn, Donna Davis, Lynda Doomy, Perrin Henderson, MS, Alicia Jessup, Kimberly Lane, Beverly Levine, PhD, Jessica McCanless, MS, Sharon McDaniel, Kathryn Melius, MS, Christine O’Neill, Angelina Pack, RN, Ritu Rathee, RN, Scott Rushing, Jennifer Sheets, Sandra Soots, RN, Michele Wall, Samantha Wheeler, John White, Lisa Wilkerson, Rebekah Wilson, Kenneth Wilson, Deb Burcombe, Georgia Saylor, Megan Lunn, Karina Ordonez, Ashley O’Steen, MS, Leigh Wagner.

Atrium Health: Michael S. Runyon, MD, MPH, Lewis H. McCurdy, MD, Yhenneko J. Taylor, PhD, Lydia Calamari, MD, Hazel Tapp, PhD, Michael Brennan, DDS, Lindsay Munn, PhD RN, Timothy Hetherington, MS, Lauren C. Lu, Connell Dunn, Melanie Hogg, MS, CCRA, Andrea Price, Marina Leonidas, Melinda Manning, Frank X. Gohs, MS, Anna Harris, MPH, Jennifer S. Priem, PhD, MA, Pilar Tochiki, Nicole Wellinsky, Crystal Silva, Tom Ludden, PhD, Jackeline Hernandez, MD, Kennisha Spencer, Laura McAlister.

Wake Med Health and Hospitals: William H. Lagarde, MD, LaMonica Daniel, BSCR.

George Washington University Data Coordinating Center: Sharon L. Edelstein, ScM, Michele Santacatterina, PhD, Greg Strylewicz, PhD, Brian Burke, MS, Mihili Gunaratne, MPH, Meghan Turney, MA, Shirley Qin Zhou, MS, Ashley H. Tjaden, MPH, Lida Fette, MS, Asare Buahin, Matthew Bott, Sophia Graziani, Ashvi Soni, MS, Guoqing Diao, PhD, Jone Renteria, MS.

George Washington University Mores Lab: Christopher Mores, PhD, Abigail Porzucek, MS.

Oracle Corporation: Rebecca Laborde, Pranav Acharya.

Vysnova Partners: Anne McKeague, PhD, Johnathan Ward, MS, Diana P. Naranjo, MA, Nana Darko, MPH, Kimberly Castellon, BS, Ryan Brink, MSCM, Haris Shehzad, MS, Derek Kuprianov, Douglas McGlasson, MBA, Devin Hayes, BS, Sierra Edwards, MS, Stephane Daphnis, MBA, Britnee Todd, BS.

## Data Availability

The data that support the findings of this study are available from the corresponding author upon reasonable request.
